# Supplementary material for: The interactome of intact mitochondria by cross-linking mass spectrometry provides evidence for coexisting respiratory supercomplexes
Source: Mol Cell Proteomics. 2017 Dec 8;17(2):216–32. doi: 10.1074/mcp.RA117.000470 (PMC5795388; doi:10.1074/mcp.RA117.000470)
Supplement: Supplemental Data [file supp_RA117.000470_133922_0_supp_23978_fzffwf.zip › spectra_annotation/mito_spectra_annotation/256-2-13-1-5-1.pdf]

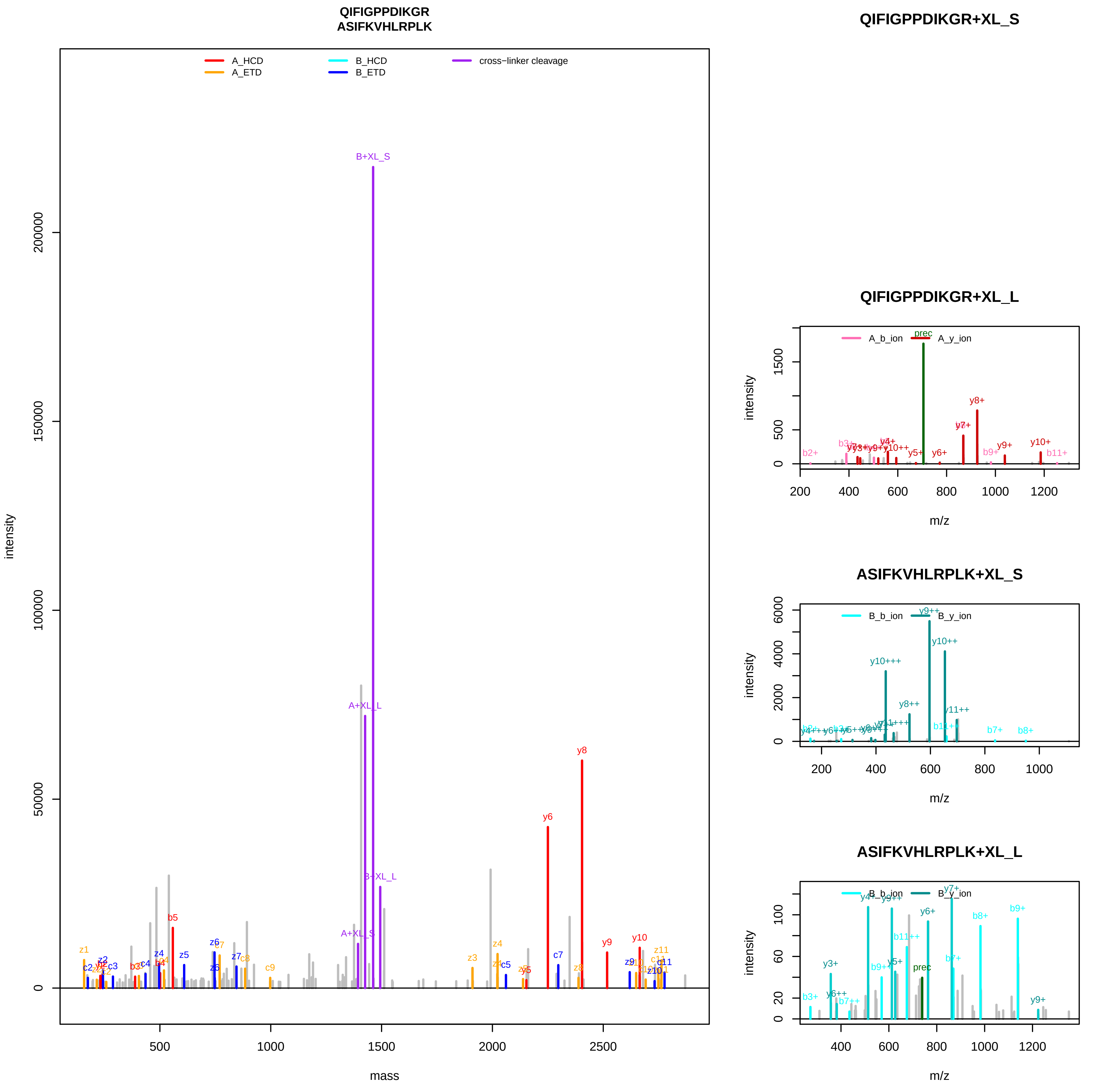
QIFIGPPDIKGR
ASIFKVHLRPLK
A_HCD
A_ETD
B_HCD
B_ETD
cross−linker cleavage
B+XL_S
A+XL_L
B+XL_L
A+XL_S
y6
y8
b5
y9
y10
z1
z3
z4
z5
z6
z7
z8
z9
z11
c2
c3
c4
c5
c7
c8
c9
c11
z10
intensity
mass
0
50000
100000
150000
200000
500
1000
1500
2000
2500
QIFIGPPDIKGR+XL_S
QIFIGPPDIKGR+XL_L
A_b_ion
A_y_ion
prec
b2+
b3+
b9+
b11+
y4+
y5+
y6+
y7+
y8+
y9+
y10+
y10++
intensity
m/z
0
500
1500
200
400
600
800
1000
1200
ASIFKVHLRPLK+XL_S
B_b_ion
B_y_ion
y9++
y10++
y10+++
y8++
y11++
b7+
b8+
intensity
m/z
0
2000
4000
6000
200
400
600
800
1000
ASIFKVHLRPLK+XL_L
B_b_ion
B_y_ion
y7+
y4+
y9++
y6+
b8+
b9+
b11++
y3+
y5+
b7+
prec
b3+
y6++
y9+
intensity
m/z
0
20
60
100
400
600
800
1000
1200
